# A Novel Top-*k* Strategy for Influence Maximization in Complex Networks with Community Structure

**DOI:** 10.1371/journal.pone.0145283

**Published:** 2015-12-18

**Authors:** Jia-Lin He, Yan Fu, Duan-Bing Chen

**Affiliations:** 1 Web Sciences Center, University of Electronic Science and Technology of China, Chengdu 611731, People’s Republic of China; 2 Big Data Research Center, University of Electronic Science and Technology of China, Chengdu 611731, People’s Republic of China; IFIMAR, UNMdP-CONICET, ARGENTINA

## Abstract

In complex networks, it is of great theoretical and practical significance to identify a set of critical spreaders which help to control the spreading process. Some classic methods are proposed to identify multiple spreaders. However, they sometimes have limitations for the networks with community structure because many chosen spreaders may be clustered in a community. In this paper, we suggest a novel method to identify multiple spreaders from communities in a balanced way. The network is first divided into a great many super nodes and then *k* spreaders are selected from these super nodes. Experimental results on real and synthetic networks with community structure show that our method outperforms the classic methods for degree centrality, *k*-core and ClusterRank in most cases.

## Introduction

Spreading process is one of the fundamental processes taking place in complex networks [1–5]. It has been applied in many fields, such as information diffusion [6], disease propagation [4], cascade failure [7], etc. Identifying a set of critical spreaders is an important issue in spreading process [8–11]. For example, in August 2003, three burned power lines in Northern Ohio brought about serious disaster that the entire US Northeast and parts of Canada were plunged into darkness. If the vulnerable regions in power-grid network are known well in advance, we could take some measures to protect them. So a set of critical spreaders is crucial for developing efficient strategies to control the spreading process in complex networks.

In the past years, some special methods have been proposed to identify multiple spreaders. Kempe et al. [12] presented a hill-climbing strategy to choose *k* spreaders. They demonstrated that the greedy strategy achieves an approximation guarantee of (1-1/*e*) where *e* is the base of the natural logarithm. Narayanam et al. [13] proposed a SPIN heuristic algorithm for the top-*k* nodes problem. To compute the Shapley values required by the SPIN algorithm, they use a simple sampling technique to obtain a computationally efficient scheme. Zhao et al. [14] made an attempt to find effective multiple spreaders in complex networks by generalizing the idea of the coloring problem in graph theory to complex networks. In their method, the nodes with the same color are sorted into an independent set. Then, for a given centrality, the nodes with the highest centrality in an independent set are chosen as multiple spreaders. Chen et al. [15] proposed degree discount heuristics, which nearly match the performance of the greedy methods for the IC model, while also improve upon the pure degree heuristic in other cascade models. Zhang et al. [16] proposed a novel method for identifying influential nodes in complex networks with community structure. The method uses the information transfer probability between any pair of nodes and the *k*-medoid clustering algorithm.

There are two benchmark methods for the identification of multiple spreaders in complex networks. The first one chooses the top *k* influential nodes as spreaders according to a centrality index [17–29]. Although the method is very simple, most of these *k* spreaders may be clustered in a community. The second one chooses *k* unconnected spreaders according to a centrality index. However, many spreaders may still locate in a community. In this paper, we suggest a novel method which disperses *k* spreaders. A network is first divide into a great many super nodes and then *k* spreaders are chosen from these super nodes according to a centrality index. If a super node includes one spreader, the nodes, which have edges incident to the super node, can not be selected as spreaders any more. The SIR model is used to test the performance of our method. Experimental results on real and synthetic networks with community structure show that our method outperforms the benchmark methods for degree centrality, *k*-core and ClusterRank in most cases.

## Materials and Methods

### Super Node

Loosely speaking, a community is a subgraph of a network whose nodes are more tightly connected with each other than with nodes outside the subgraph. Usually, a community exhibits hierarchical organization, that is, it can contain groups of sub-communities, and so forth over multiple scales. [30]. The community hierarchy can be found by Blondel method [31], which is composed of two steps. In the first step, each community adjusts their nodes according to the increment of modularity. In the second step, each community is replaced by a new node called “super node”. The two steps are repeated until the modularity can not be improved. In this paper, to obtain a great many communities, the two steps are iterated only once.

### Red-Black Tree

The red-black tree [32] is a type of binary search tree where costs are guaranteed to be logarithmic, no matter what sequence of keys is used to construct them. In the tree, each node is either red or black. It has perfect black balance, i.e., every path from the root to a null link contains the same number of black nodes. The average length of a path from the root to a node in a red-black tree with *n* nodes is approximately equal to log *n*. So in a red-black tree, searching operation, insertion operation or ranking operation takes only logarithmic time in the worst case.

### Spreader Identification

All super nodes are stored in a red-black tree. For a super node, the key is its id and the values contain its size and its nodes. Besides, it contains a state variable which indicates whether the super node is visited. We first take a non-visited super node with maximal size from the red-black tree. Then we select the most influential node from the super node as a spreader according to a centrality index. Similarly, we take the next super node from the red-black tree and select the most influential node as a spreader, which has no edges incident to the super nodes which have already contained spreaders. If all super nodes are visited and the number of chosen spreaders is not enough, we restart to visit all super nodes in the descending order of their size and choose the remaining spreaders. The process is repeated until *k* spreaders are found. In practice, the number of super nodes is far more than that of the spreaders. So *k* spreaders can be always identified in the first sweep.

**Fig 1:**
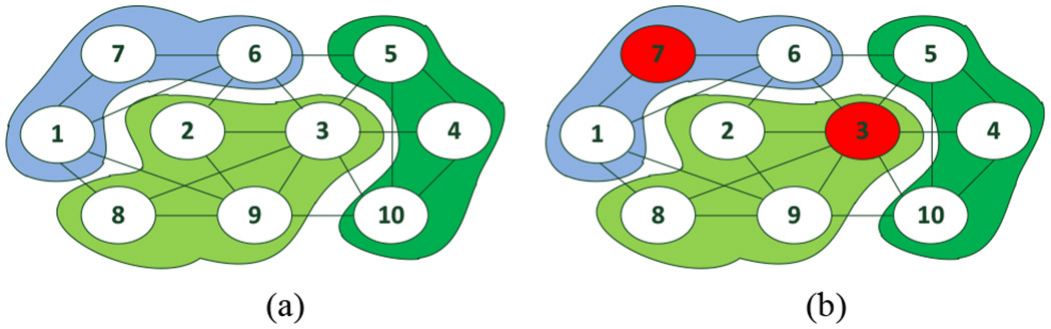
The spreader identification process of our method. (a) A toy network with 10 nodes and 3 super-nodes; (b) two spreaders identified by our method.

In Fig 1, we use a toy network with 10 nodes and 3 super nodes to illustrate our method. Two spreaders will be chosen from the network and degree centrality is used to measure the influence of each node. As shown in Fig 1(a), three super nodes are represented by three different colors respectively. For the biggest super node (2,3,8,9), node 3 is the most influential node and is chosen as a spreader. Nodes 1,4,5,6 and 10 can not be selected as spreaders because they have at leat one edge incident to the super node (2,3,8,9). Node 7 is chosen as the second spreader and the final result is shown in Fig 1(b).

### Computational Complexity

The computational complexity of our method is analyzed as follows. The super nodes can be found in *O*(*m*) time by using the Blondel method, where *m* is the number of edges in network. Since an insertion operation in a red-black tree with *r* super nodes takes *O*(log *r*) time, so the construction of a red-black tree with *l* super nodes takes *O*(log (*l* − 1)!) < *O*(*l*log *l*) time. A searching operation in a red-black tree is guaranteed to visit at most log *l* nodes, so *k* visits totally take *O*(*k*log *l*) time. Finally, identifying a spreader in a super node takes *O*(*s*) in the worse case, where *s* is the size of the super node and identifying *k* spreaders totally take *O*(*n*) time. So the total running time of our method is *O*(*m* + *n* + (*k* + *l*)log *l*).

## Results

We simulate the spreading process in a network by using the SIR model [33] which has been extensively studied. In the SIR model, each node has one of three states (Susceptible, Infected and Recovered) at each time step. An infected node randomly contacts a neighbor node and transmits the disease to it with a probability *μ* if the neighbor node is a susceptible one. At the same time, an infected node will be recovered with a probability *β*. The effective spreading rate *λ* is defined as *μ*/*β*. When there is no infected nodes in a network, the spreading process stops.

### Real Networks

The performance of our method is evaluated on three real networks, including Gowalla, Dblp and Youtube networks. Gowalla network [34] contains user-user friendship relations. Nodes represent users and an edge indicates a friendship between two users. Dblp network [35] is a co-authorship network from computer science bibliography. Nodes represent authors and an edge between two nodes exists if two corresponding authors have published at least one paper together. Youtube network [35] is a social network from a video-sharing web site. Users form friendships with each other and users can create groups in which other users can join. In the network, nodes represent users and an edge between two nodes indicates a friendship. The detailed information of the three real networks is listed in Table 1.

**Table 1:** The topological properties of three real networks, including the number of nodes, the number of edges, the number of super nodes, average degree (<*k*>), modularity (Q), mean squared degree (<*k* ^2^>) clustering coefficient (cc), power law exponent (*α*) and maximal *k*-core value (*k*-core).

network	#nodes	#edges	#super-nodes	<*k*>	Q	<*k* ^2^>	cc	*α*	*k*-core
Gowalla	196591	950327	21954	9.67	0.63	2964.03	0.024	2.65	51
Dblp	317080	1049866	63479	6.62	0.58	144.01	0.306	3.26	113
Youtube	1134890	2987624	172928	5.26	0.61	2603.72	0.006	2.14	51

We compare our method (labeled as super-node) with two benchmark methods on three real networks. The first method (labeled as influential-node) chooses the top *k* influential nodes as spreaders according to a centrality index. The second method (labeled as disperse-node) first computes a ranking list of nodes based on a centrality index and then selects *k* unconnected spreaders from the ranking list. Three centrality indices, i.e., degree centrality, *k*-core and ClusterRank, are chosen to measure the influence of each node in network.

**Fig 2:**
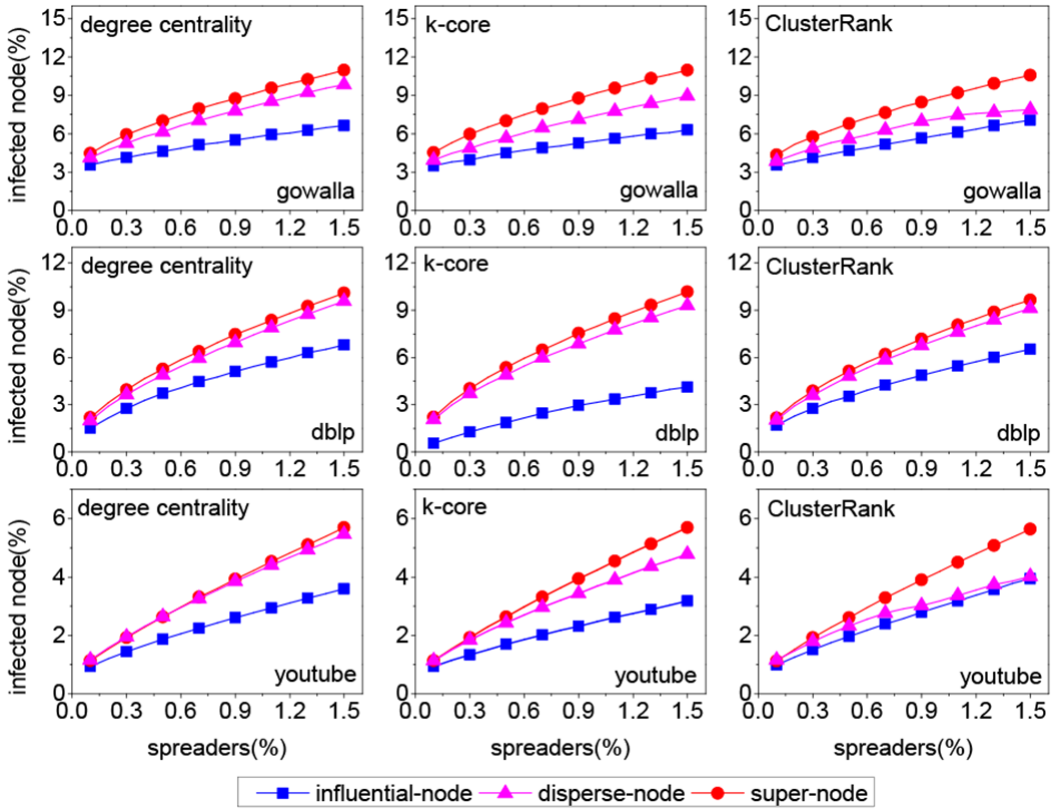
The influence scope with different proportions of spreader on three real networks, where *λ* = 1.5, *β* = 1/<*k*>. Each data point is obtained by averaging over 200 independent runs.

**Fig 3:**
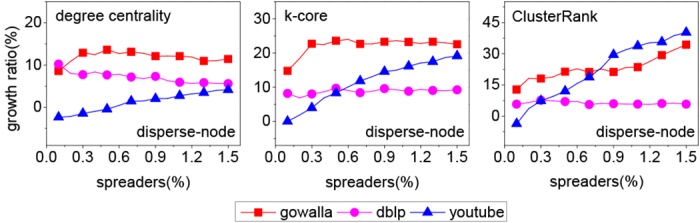
The growth ratio on three real networks for three centrality indices.

From Fig 2, it can be seen that both our method and the disperse-node method outperform the influential-node method greatly in most cases. So the following analysis only involves our method and the disperse-node method. To quantify the performance of two methods, we define an index called “growth ratio”,

$$\begin{array}{c} growth\;ratio=\frac{p_{our\;method}-p_{other\;method}}{p_{other\;method}}, \end{array}$$ (1)

where *p*_*our method*_ is the proportion of infected nodes in a network for our method and *p*_*other method*_ for benchmark method. Fig 3 shows that our method influences a greater scope than the disperse-node method in most cases. It is noted that the growth ratio is related to network structure. All growth ratios for Dblp network are low and most of them are less than 10%. However, for the other two networks, most of growth ratios are above 10% and the maximum is more than 30%. Meanwhile, the growth ratio has also to do with centrality index. For degree centrality, the growth ratios are less than 20% on three networks. For *k*-core, most of growth ratios are more than 20% on Gowalla network. For ClusterRank, most of growth ratios are more than 30% on Gowalla and Youtube networks.

To further evaluate the performance of our method, we compare it with the *k*-medoid method [16], which also chooses *k* spreaders from communities. In the *k*-medoid method, each edge(*u*, *v*) is randomly designated either “open” with probability *β*_*uv*_ or “closed” with probability 1-*β*_*uv*_ independently. The *β*_*uv*_ is defined as

$$\begin{array}{c} \beta_{uv}=1-{\left(1-\beta \right)}^{w_{uv}}, \end{array}$$ (2)

where *w*_*uv*_ is the weight of edge(*u*, *v*) and *β* is a designated propagation probability. For two nodes *p* and *q*, if there is at least a path between them which is composed of “open” edges, *ω*(*p*, *q*) = 1, otherwise 0. Then the element *m*_*pq*_ of information transfer probability matrix *M* is defined as

$$\begin{array}{c} m_{pq}=\frac{1}{N}\sum_{i=1}^{N}\omega \left(p,q\right), \end{array}$$ (3)

where *N* is the number of sampling. The network is first divided into *k* communities based on *M* and then *k* medoids are chosen as *k* spreaders. In the *k*-medoid method, the time complexity of each iteration is *O*(*k*(*n* − *k*)^2^), where *n* is the number of nodes in network. So the method is very time consuming.

Because of high time complexity, the *k*-medoid method can not be applied to Gowalla, Dblp and Youtube networks. So two small real networks, i.e., karate [36] and football networks [37], are used in this experiment. Karate network reflects the social relations of a karate club in an American university. Its nodes represent club members, and an edge indicates social communication between two club members. It includes 34 nodes and 78 edges. Football network is the match network of American football games between Division IA colleges during regular season Fall 2000. Its nodes represent teams, and an edge indicates that a match is played between the two corresponding teams. It contains 115 nodes and 613 edges. The detailed information of the two real networks is described in Table 2.

**Table 2:** The topological properties of two real networks, including the number of nodes, the number of edges, the number of super nodes, average degree (<*k*>) and modularity (Q).

network	#nodes	#edges	#super-nodes	<*k*>	Q
Karate	34	78	7	4.59	0.37
Football	115	613	13	10.67	0.58

**Fig 4:**
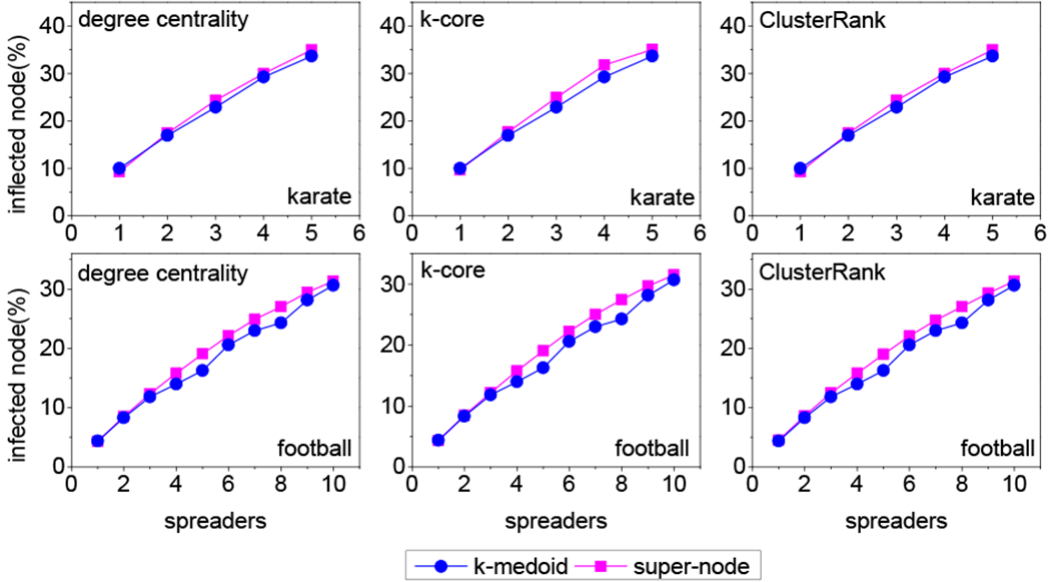
The comparisons between our method and the *k*-medoid method on karate and football networks, where *λ* = 1.1, *β* = 1/<*k*>. Each data point is obtained by averaging over 100000 independent runs.

From Fig 4, it can be seen that two methods have approximate performance. However, in most cases, our method outperforms the *k*-medoid method slightly. Besides, compared with the *k*-medoid method, our method has two advantages. First, the *k*-medoid method must divide a network into *k* communities to choose *k* spreaders. However, the *k* communities may not meet the community definition, that is, the nodes are denser within communities than across. For our method, the detected communities correspond to the real communities in network because the Blondel method is employed. Second, it is difficult to apply the *k*-medoid method to large networks because of high time complexity. Conversely, our method can choose *k* spreaders quickly in large networks because of low time complexity.

### Synthetic Network

We also test the performance of our method on three synthetic scale-free networks which are generated by LFR model [38]. In the LFR model, both the degree and the community size distributions are power laws, with exponents *α* and *β*, respectively. In our experiment, three synthetic networks have the same parameters *α* and *β*, which are set to 2.5 and 2.5 respectively. The only difference for three synthetic networks is the mixing parameter *μ*, which is set to 0.1, 0.3 and 0.5 respectively. The detailed information of the three synthetic networks is described in Table 3.

**Table 3:** The topological properties of three synthetic networks, including the number of nodes, the number of edges, minimum degree (*k*_*min*_), average degree (<*k*>), the number of super nodes and modularity (Q).

network	#nodes	#edges	*k*_*min*_	<*k*>	*α*	*β*	*μ*	#super-nodes	Q
LFR1	10000	65338	5	13.07	2.5	2.5	0.1	154	0.74
LFR2	10000	65338	5	13.07	2.5	2.5	0.3	225	0.57
LFR3	10000	65338	5	13.07	2.5	2.5	0.5	716	0.35

**Fig 5:**
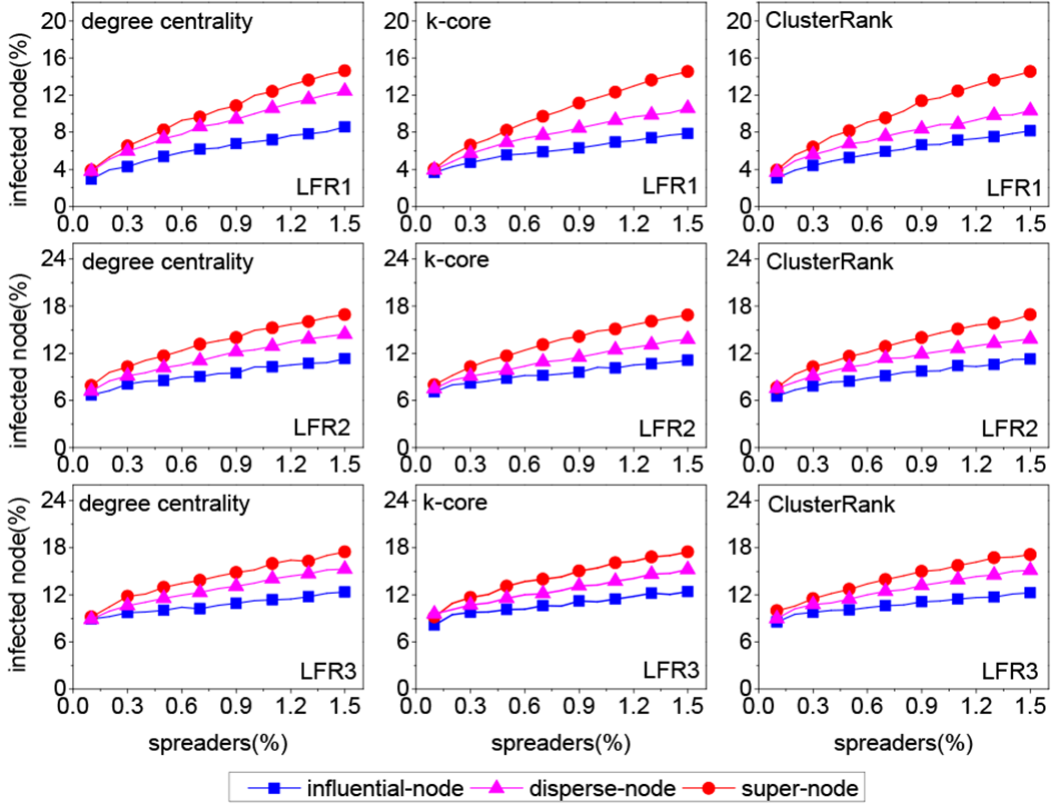
The influence scope with different proportions of spreader on three synthetic networks, where *λ* = 1.5, *β* = 1/<*k*>. Each data point is obtained by averaging over 200 independent runs.

**Fig 6:**
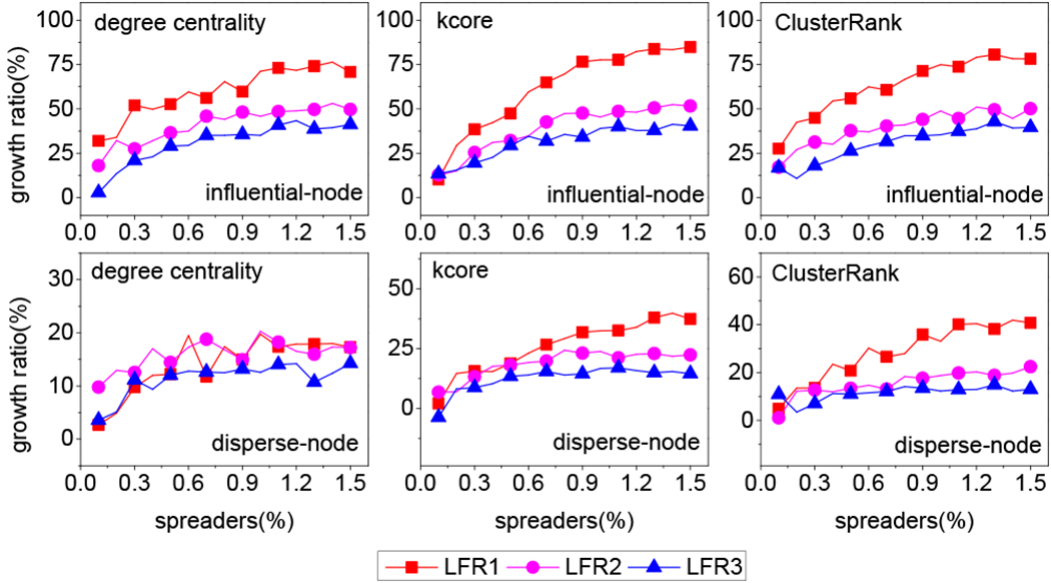
The grow ratios on three synthetic networks.

**Fig 7:**
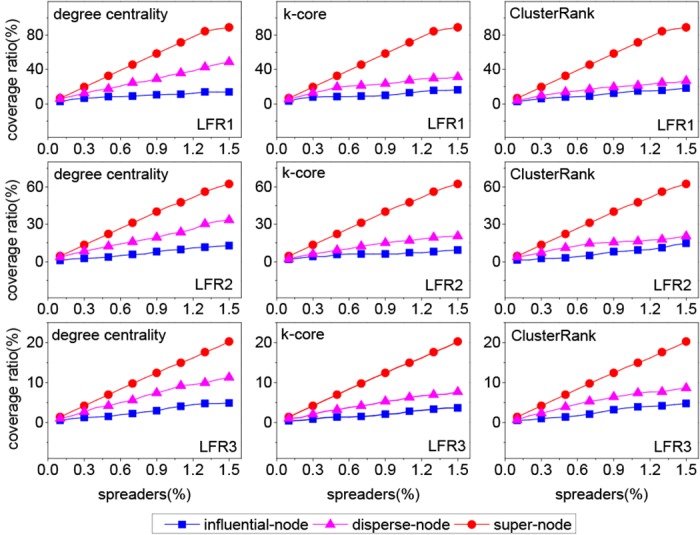
The coverage ratios on three synthetic networks.

From Fig 5, it can be seen that our method outperforms two benchmark methods in most cases. The corresponding growth ratio is shown in Fig 6. In most cases, the growth ratio is the highest for the LFR1 network and the lowest for the LFR3 network. Interestingly, the modularity of the LFR1 network is the highest and that of the LFR3 network is the lowest, as shown in Table 3. So the growth ratio is proportional to the modularity of network in most cases. The reason can be explained from two aspects, i.e., the structure of super node and the dispersion degree of *k* spreaders. First, the higher the modularity is, the denser the structure of the super node is. If two or more spreaders locate in a dense super node, they have many common neighbors. Once the common neighbors are infected, these spreaders have less chances to contact susceptible nodes at each time step. Second, to quantify the dispersion degree of *k* spreaders, we define an index named “coverage ratio”,

$$\begin{array}{c} coverage\;ratio=\frac{＃{super-node}^{\prime }}{＃super-node}, \end{array}$$ (4)

where #super-node is the number of all super nodes in network and #super-node’ is the number of super nodes which contain at least one spreader in network. As shown in Fig 7, the coverage ratio of our method is higher than that of two benchmark methods. Take the LFR1 network for example, the coverage ratio is more than 80% for our method, less than 50% for the disperse-node method and less than 20% for the influential-node method. So compared with two benchmark methods, our *k* spreaders are more disperse. In fact, for our method, a super node usually contains at most one spreader because the number of super nodes is far more than that of the spreaders. However, for two benchmark methods, many super nodes contain two or more spreaders. From the above analysis, our method is suitable for the networks with obvious community structure.

## Discussion

In this paper, we suggest a novel top-*k* strategy which chooses multiple spreaders from communities. In our method, the network is first divided into many super nodes and then *k* spreaders are selected from these super nodes. If a super node contains one spreader, the nodes, which have at least one edge incident to the super node, are not chosen as spreaders any more. In practice, the number of super nodes is far more than that of spreaders, so a super node usually contains at most one spreader.

The performance of our method is evaluated on real and synthetic networks with community structure. On three large real networks, our method outperforms two benchmark methods in most cases. The growth ratio is not only related to network structure but also has to do with centrality index. On two small real networks, our method outperforms the *k*-medoid method slightly in most cases. Compared with the *k*-medoid, our method has two advantages. First, the detected communities correspond to the real communities in network. Second, the time complexity is low. On three synthetic scale-free networks, our method still outperforms two benchmark methods in most cases. Compared with two benchmark methods, our method has more chances to contact susceptible nodes on the synthetic network with high modularity.

There are two open issues needing further study in the future. First, the performance of our method is related to centrality index. So how the centrality index affects the identification of multiple spreaders should be studied. Second, with the available of temporal data in recent years, the spreading process in temporal networks has caused great concern [39, 40]. So the further research on the spreader identification in temporal networks is needed.
