# Correction: A Novel Top-*k* Strategy for Influence Maximization in Complex Networks with Community Structure

**DOI:** 10.1371/journal.pone.0162066

**Published:** 2016-08-25

**Authors:** Jia-Lin He, Yan Fu, Duan-Bing Chen

The Funding statement appears incorrectly in the published article. The correct Funding statement is: This work is partially supported by the National Natural Science Foundation of China (http://isisn.nsfc.gov.cn/egrantindex/funcindex/prjsearch-list#) under Grant No. 61433014, and by the Fundamental Research Funds for the Central Universities (http://isisn.nsfc.gov.cn/egrantindex/funcindex/prjsearch-list#) under Grant No. ZYGX2014Z002. The funders had no role in study design, data collection and analysis, decision to publish, or preparation of the manuscript.
